# Monoclonal Antibody-Based Immunotherapy and Its Role in the Development of Cardiac Toxicity

**DOI:** 10.3390/cancers13010086

**Published:** 2020-12-30

**Authors:** Mohit Kumar, Chellappagounder Thangavel, Richard C. Becker, Sakthivel Sadayappan

**Affiliations:** 1 Heart, Lung and Vascular Institute, Department of Internal Medicine, Division of Cardiovascular Health and Disease, University of Cincinnati, Cincinnati, OH 45267, USA; beckerrc@ucmail.uc.edu (R.C.B.); sadayasl@ucmail.uc.edu (S.S.); 2 Department of Radiation Oncology, Sidney Kimmel Medical College, Thomas Jefferson University, Philadelphia, PA 19107, USA; thangavel.chellappagounder@jefferson.edu; 3 Department of Dermatology, Sidney Kimmel Medical College, Thomas Jefferson University, Philadelphia, PA 19107, USA

**Keywords:** cardiomyocyte, heart failure, heart failure with preserved ejection fraction (HFpEF), immune checkpoint inhibitors, cardiotoxicity

## Abstract

**Simple Summary:**

The application of immunotherapies to treat cancer patients has significantly improved over the last two decades and extended many patients’ life spans. Monoclonal antibodies are synthetic proteins employed as immunotherapies to treat and manage cancers that require a complete understanding of cancer biology and the host’s immune system. However, activated immune responses, by monoclonal antibodies, can target nonspecific cancer cells, causing frequent immune-related adverse events that can lead to permanent disorders among cancer patients. The immune-related adverse events pose a risk of cardiac toxicity that includes hypertension, heart failure, arrhythmias, and left ventricular dysfunction during and after monoclonal antibody immunotherapy. Moreover, with the mortality rate of 47% attributed to heart disease and cancer, it is imperative to employ reliable, sensitive, and clinically relevant models for efficacy and safety assessment of immune drugs for cancer and the prevention of cardiotoxicities.

**Abstract:**

Immunotherapy is one of the most effective therapeutic options for cancer patients. Five specific classes of immunotherapies, which includes cell-based chimeric antigenic receptor T-cells, checkpoint inhibitors, cancer vaccines, antibody-based targeted therapies, and oncolytic viruses. Immunotherapies can improve survival rates among cancer patients. At the same time, however, they can cause inflammation and promote adverse cardiac immune modulation and cardiac failure among some cancer patients as late as five to ten years following immunotherapy. In this review, we discuss cardiotoxicity associated with immunotherapy. We also propose using human-induced pluripotent stem cell-derived cardiomyocytes/ cardiac-stromal progenitor cells and cardiac organoid cultures as innovative experimental model systems to (1) mimic clinical treatment, resulting in reproducible data, and (2) promote the identification of immunotherapy-induced biomarkers of both early and late cardiotoxicity. Finally, we introduce the integration of omics-derived high-volume data and cardiac biology as a pathway toward the discovery of new and efficient non-toxic immunotherapy.

## 1. Introduction

Approximately 47% of all mortality can be attributed to heart disease and cancer in the United States [1]. Traditional chemotherapies can result in cardiotoxicity, as manifested by arrhythmias and heart failure [2,3]. The emerging field of onco-immunology has led to the development of monoclonal antibodies (mAbs) to promote antitumor T-cell responses and response factors by inhibiting negative regulatory proteins on T-cells. This specific class of mAbs, known as immune checkpoint inhibitors (ICIs), targets cytotoxic T-lymphocyte antigen-4 (CTLA-4), programmed cell death protein-1 (PD-1), and programmed cell death ligand-1 (PD-L1). Anti-PD-1/PD-L1 mAbs are widely administered as single agents or fused with other therapeutic agents to boost T-cell function and destroy cancer cells in 50 different cancer types [4].

**Figure 1:**
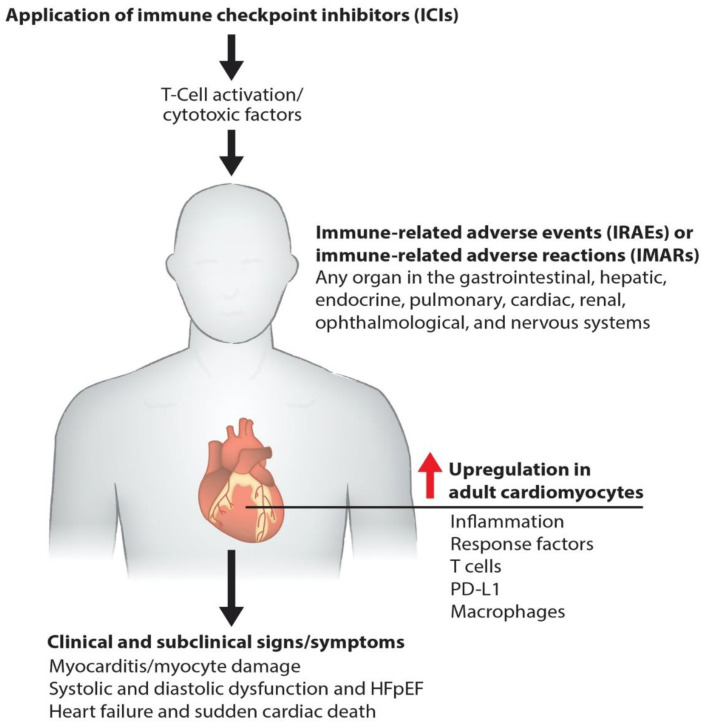
Molecular pathogenesis of immunotherapy-induced cardiac toxicity. Schematics describe how the ICIs such as anti-PD-1, anti-PD-L1, and anti-CTLA-4 promote myocarditis via cardiac inflammation, causing myocyte damage. Cardiac myocyte dysfunction impairs systolic and diastolic function and causes failure and death.

Immunotherapies pose a risk of cardiac toxicity, including hypertension, heart failure, QT interval prolongation, left ventricular dysfunction (LVD), and heart failure with preserved ejection fraction (HFpEF), during and after therapy [5,6,7], as shown in our model (Figure 1). Pathogenic cardiotoxicity is also associated with other risk factors, including age, drug dosage, and pre-existing cardiovascular disease. A clear understanding of the interaction between cancer therapies and the cardiovascular system at the molecular level can facilitate the early detection and prevention of cardiotoxicity. In this review, we discuss immunotherapy known to cause cardiovascular toxicity. We also outline novel platforms and strategies for investigating cardiovascular toxicity by employing human-induced pluripotent stem cell-derived cardiomyocytes (hiPSC-CMs) and cardiac organoids, along with traditional biomarker detection and cardiovascular imaging. 

## 2. Contemporary Cancer Immunotherapy

Simply stated, immunotherapies are introduced to either activate or suppress the immune system. This form of treatment is the product of several decades of cancer research, together with an increased understanding of antitumor immune responses. A major advance came in the form of immune checkpoint receptor biology and its importance in determining host responses to cancer [8,9,10]. Several different immunotherapy strategies are currently available for patient care, such as adoptive immune cell therapies, immune modulators (ICIs, interleukins, interferons), targeted monoclonal antibodies, and cancer vaccines. Adoptive cell therapy (ACT) is a novel and promising method involving the direct intervention of a patient’s immune system to combat a broad spectrum of cancer cells [5,11]. For example, natural killer T-cells bind antigens on the surface of cancer cells. Several ACT immunotherapies take advantage of this natural ability and are employed in Tumor-Infiltrating Lymphocyte Therapy (TILT), Engineered T-Cell Receptor Therapy (ETCR), Chimeric Antigen Receptor T-Cell Therapy (CARTCT), and Natural Killer Cell Therapy. However, “killer” T-cells once activated and remain viable in large numbers for a sufficiently long time to mount an effective antitumor response. In TILT, naturally occurring T-cells are collected, genetically activated, and then expanded with interleukin-2. Next, cells are reinfused into the patients’ tumor microenvironment (TME) to kill tumor cells. In some patients, these engineered T-cells can also be reinfused with a new T-cell receptor that allows them to target specific cancer antigens. Similarly, patients’ T-cells can be infused with the synthetic chimeric antigen receptor (CAR), which helps treat patients with certain types of large B-cell lymphoma [12].

**Figure 2:**
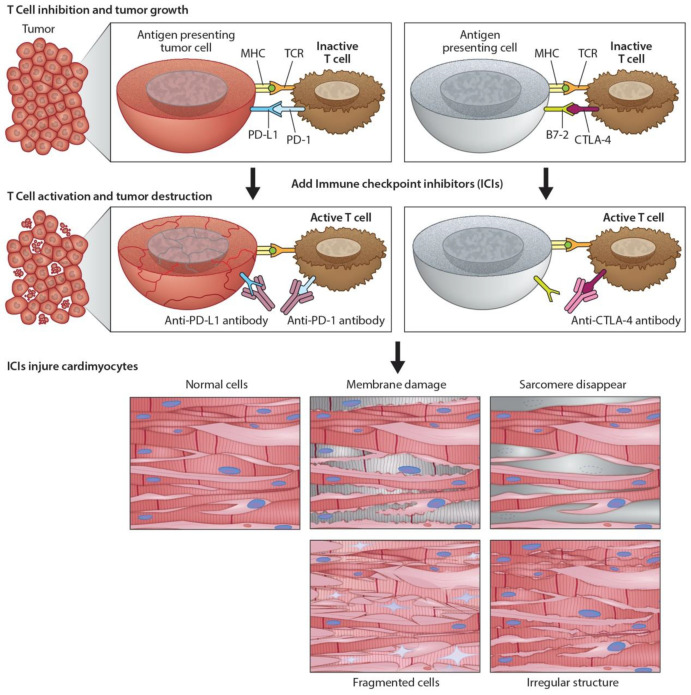
Programmed death ligand-1 signaling and CTLA-4 deregulation facilitates tumor growth. Cartoon illustrates how PD-L1 interacts with PD-1 to inhibit T-cell activation and promote tumor cell proliferation (top panel). Working model of ICIs and promotion of cardiotoxicity (bottom panel). Cartoon illustrates how monoclonal anti-PD-1, anti-PDL-1, and anti-CTLA-4 activate T-cells following their binding to respective ligands. The activated T-cells kill/destroy the tumor cells by producing cytotoxic effects on cancer cells. In addition to tumor suppression, ICIs also promote cardiac myocyte damage, impairing function.

Immunomodulators use small molecules to regulate the immune system and immune response to tumorigenesis [13]. Tumors can disable regulatory immune checkpoints to shut down immune responses, protect themselves, and proliferate. In particular, tumor cells use T-cell receptors and PD-1 protein to avoid destruction by natural killer cells. In response, checkpoint inhibitors [4] can act as antibodies against immune checkpoint receptors to bind and block the tumor’s inhibitory signaling downstream from CTLA-4 and PD-1, the host immune down-regulation receptors. This results in unleashing immune responses, as well as enhancing existing cytotoxic host immune response to the cancer cells (Figure 2). Cancer vaccines [14] allow the immune system to identify tumorous threats according to their specific antigens and then mount the proper response. Each patient’s tumor has a unique microenvironment, or TME, that expresses its own antigens to help distinguish cancer from normal cells. These antigens can be normal proteins produced in large numbers, or they can be new proteins because of mutations and expressed exclusively by tumor cells that become the target for the directed vaccine response. 

## 3. Monoclonal Antibodies (mAbs) and Their Application in Cancer Immunotherapy

Clinical application of mAbs immunotherapy to treat and manage many cancers has been successfully established in recent years [10]. Primary solid tumors have been effectively treated and managed with antibody-chemotherapy conjugates [10]. The development and establishment of therapeutic monoclonal antibodies require a complete understanding of tumor heterogeneity, intra-tumoral factors, protein engineering, and the interaction between cancer cells and the immune system. Researchers have now developed mAbs that target specific antigens in cancer cells and inhibit signals responsible for tumor growth and invasion [15]. Monoclonal antibodies are simply synthetic proteins employed as immunotherapies to treat and manage some, but not all, cancers. Monoclonal antibodies are produced in four different forms, including murine, chimeric, humanized, and human. 

### 3.1. Development of Therapeutic mAbs

The production of mAbs uses hybridoma by coupling myeloma cells with desired antibody-producing B-cells, typically from murine protein sources (-omab) [8,16]. From hybridoma, clones based on antigen specificity and immunoglobulin class are screened and selected to produce a single epitope. However, mAbs derived from mice are restricted owing to the generation of the innate immune response in humans. Therefore, steps were taken to generate humanized antibody by chimeric fusion of part mouse and part human proteins (-ximab), by a small portion from mouse and a major portion of protein from human (-zumab), or fully human antibody by using only human proteins (-umab) to overcome deficiencies in the mouse immune system. Around 5% of humanized mAbs contains mouse-derived antigen-binding factor, and this portion of humanized mAbs is generally engineered from human sources. However, mAbs generated from hybridomas of human or humanized mouse origin systems using high-throughput immunoassays have the strongest affinities to antigens [17].

### 3.2. Types of mAbs

Three types of monoclonal antibodies are employed in clinics to treat cancer patients to boost their immune system includes (1) Naked mAbs, (2) Conjugated mAbs, and (3) Bispecific mAbs. Naked mAbs are not conjugated with drug or radioactive material [18]. Conjugated mAbs are radiolabeled and chemolabeled antibodies [19]. Bispecific mAbs consist of parts of two different binding fragments of Abs, attaching two different proteins and bringing two cells in proximity to one another. 

### 3.3. Mechanism of Action of mAbs in Cancer Treatment

Monoclonal antibodies target cancer cells in different and unique ways [7,9,20,21]. Naked antibodies function by themselves without any type of conjugated drug or radioactive materials. Most naked mAbs bind to antigen-bearing cancer cells, healthy cells, and free-floating proteins. Naked mAbs work by boosting the host immune system against cancer cells by binding to an antigen-presenting cancer cell. For example, alemtuzumab binds to the CD52 antigen in chronic lymphocytic leukemia patients and attracts immune cells to destroy lymphocytes. Alternatively, naked mAbs bind to immune checkpoint proteins, thereby stimulating the host immune response. The drugs pembrolizumab, Nivolumab, and Cemiplimab bind to PD-1 protein, while Atezolizumab, Avelumab, and Durvalumab bind to PD-L1 proteins to inhibit the evasion of cancer cells from host immune response. CTLA-4, also known as CD152, is a protein receptor that mediates immune checkpoint function and down-regulates immune responses. The mAb drug conjugate Ipilimumab binds to CTLA-4, blocking the inhibitory signal, facilitating the killing of tumor cells by cytotoxic T lymphocytes. In contrast, conjugated (tagged, labeled, or loaded) mAbs are combined with a chemotherapeutic drug or radioactive elements and function as a homing signal to deliver these drugs, or elements, directly to the cancer cells. An interesting example of a similar mechanism of action is the mAb drug trastuzumab that inhibits breast and stomach cancer cell proliferation by binding with HER2. An example of a radiolabeled antibody is ibritumomab tiuxetan which binds to the CD20 antigen on the surface of B-cells and delivers both drug and radioactivity to kill specific cancer cells. Another example of chemolabeled mAbs is brentuximab vedotin. It binds to the CD30 antigen on the surface of B-cells and attaches to a chemotherapeutic drug called monomethyl auristatin E. The bispecific mAb blinatumomab binds to CD19 protein on lymphoma cells and CD3 protein on immune T-cells, thus triggering T-cell cytotoxicity against leukemic B-cells.

### 3.4. Monoclonal Antibodies (mAbs) in Cancer Treatment and the Development of Cardiac Toxicity

Myocarditis is an inflammation of the heart muscle. ICIs causes myocarditis with the signs of dyspnea, fatigue, chest pain, palpitation, peripheral edema, or hypotension with elevated electrocardiogram and cardiac troponin (cTn) and sometimes no symptoms [22,23]. Similarly, ICIs can also cause inflammation of the pericardium, such as pericardial disease and pericarditis [24]. Monoclonal anti-CTLA-4 and PD-L1 are two major ICIs in immunotherapy. Monoclonal antibodies against PD-1 and PD-L1 are also an interventional therapy, as described in Table 1 and Figure 2 [25]. All immunotherapies have adverse side effects since activated immune responses can target nonspecific cancer cells, leading to frequent immune-related adverse events (IRAEs) or immune-related adverse reactions (IMARs) [6,26,27,28,29]. IRAEs can involve any organ in the gastrointestinal, hepatic, endocrine, pulmonary, cardiac, renal, ophthalmological, and nervous systems. IRAEs typically have delayed onset with prolonged low-grade symptoms that are, for the most part, treatable and reversible. However, some IRAEs can lead to permanent disorders among cancer patients. Monoclonal antibodies against CTLA-4, PD-1, and PD-L1 trigger IRAEs involving single organ systems, but they can simultaneously affect multiple organs in approximately <1% of patients [2]. The most common checkpoint-inhibitor-associated IRAEs are observed in the skin (34% of patients) with a rash over 30% of the patient’s body. IRAEs can also affect the gastrointestinal tract, resulting in diarrhea or colitis occurring in 13% of cancer patients. Cardiotoxicity is rare, occurring in 0.04% to 1.14% of patients receiving immunotherapy. However, potentially fatal myocarditis and arrhythmias have been reported with a significantly higher associated mortality of 25% to 50%, indicating that cardiotoxicity must be a diagnostic/prognostic consideration as these therapies expand to meet the demand [30,31]. A meta-analysis for lung cancer that included 22 clinical trials of ICIs showed the incidence of other cardiovascular toxicities, including pericardial disease, myocardial infarction, stroke, cardiac failure, and cardiorespiratory arrest, ranging from 0.7% to 2.0%. Both cancer cells and cardiomyocytes express PD-L1, ICIs bind to cancer cells and as well as to non-target cardiomyocytes. ICIs bind to cardiomyocytes and modulates immune function, and promote muscle inflammation (autoimmune myocarditis) in heart muscle [32,33]. Additionally, ICIs can induce left ventricular hypertrophy and cause cardiac dysfunction [33,34,35,36]. However, the molecular and cellular changes during the development of cardiac dysfunction are yet to be systematically studied. Similarly, the mechanism underlying autoimmune myocarditis is not very clear. However, a shared antigen between tumor cells and cardiomyocytes could become the target for activated T-cells, leading to myocardial lymphocytic infiltration showing clinical manifestation of heart failure (HF) and cardiac conduction abnormalities [7]. PD-1 and PD-L1 are expressed in mouse and human cardiac myocytes. However, inhibiting CTLA-4 and deleting PD-l have led to autoimmune myocarditis, and several cases of HF have been reported in melanoma cancer patients treated with checkpoint inhibitors [25]. In a PD-1 knockout mouse study, an increase in autoimmune response was responsible for decreased left ventricular systolic function and wall thinning with dilated right ventricles, leading to increased mortality [17]. Moreover, diffused Immunoglobulin G antibody deposition was observed in cardiomyocytes of PD-1 knockout mice, suggesting an autoimmune response to the heart. 

### 3.5. Cardio-Oncology: Clinical Presentation, Diagnosis, and Management of Cardiotoxicity

Improvement in the mortality rate among cancer patients reflects better and more precise therapies. However, such therapies have also potentially increased risk factors for the cardiovascular system, resulting in long-term clinical adverse events following initial treatment. Current advancements in cardio-oncology involve highly specialized services for both cardiac health and cancer diseases. The National Institutes of Health have funded initiatives to study cardio-oncology that focus on (1) assessing cardiovascular (CV) clinical risk factors and presentation of clinical symptoms before and during cancer therapy, (2) introducing several precise cardioprotective interventions during cancer treatment, (3) balancing risk and reward as part of prescribing immunotherapy, and (4) managing post-treatment cardiotoxicity [27].

The clinical presentation of cardiotoxicity associated with immunotherapies has a spectrum of mild nonspecific symptoms to severe disease with symptoms of overt acute HF that requires inotropic support [43]. The reported clinical symptoms can be classified according to the recommendations of the American Society of Clinical Oncology clinical practice guidelines for the management of IRAEs. Life-threatening end-stage HF is the most reported contraindication in the literature, with most patients presenting a clinical syndrome of cardiogenic shock accompanied by severe conduction abnormalities, such as advanced atrioventricular blockage or ventricular tachycardia. The moderate clinical spectrum of disease ranges from chest pain, dyspnea, fatigue, peripheral edema, bilateral rales, and syncope to paroxysmal nocturnal dyspnea and palpitations [5,44]. Such clinical spectrum highlights the necessity of assessing CV risks in cancer patients to avoid fatal cardiotoxicity following the discontinuation of immunotherapy. It also provides a rationale for using immunosuppressive therapies to avoid such unnecessary discontinuation of effective anticancer treatments [31]. These assessments can also recognize early symptoms of hemodynamic instability that can persist during or after treatment.

Providing interventions during immunotherapy strongly depends on the diagnosis of the standard cardiac clinical manifestations as described above. Elevated serum troponin T levels are used to assess both prognosis and diagnosis of major adverse cardiovascular events, such as cardiovascular-related death, cardiogenic shock, cardiac arrest, or complete heart blockage as seen in myocarditis associated with immunotherapy. Similarly, along with the use of troponin, natriuretic peptides have also been proposed in screening and surveillance for higher-risk patients in the setting of immune checkpoint inhibitor-related myocarditis. Several professional societies, including the American Society of Clinical Oncology [45], Heart Failure Association of the European Society of Cardiology [46], European Society of Medical Oncology [47], the American Society of Echocardiography, and the European Association of Cardiovascular Imaging [48], have outlined guidance for an integrated approach combining ECG, echocardiography, and biomarkers in predicting cancer immunotherapy-related cardiac failure [49,50]. However, all these suggestions are based on anecdotal data since the available peer-reviewed literature is limited, and clinicians have reached no consensus about the timing of cardiotoxicity onset, thus hindering accurate clinical diagnosis. Therefore, we look to more standardized patient profiling against to apply triple diagnostic scheme, e.g., before, during, and after immunotherapy, as follows: (1) definite symptoms, including abnormal echocardiography, positive biomarker and positive ECG; (2) probable clinical symptoms; and (3) possible symptoms with elevated biomarkers, but normal hemodynamic functions [2,48]. Immunosuppression is the major course of action to reduce drug-mediated cardiotoxicity with a high dose of prednisone [6]. Higher doses of steroids have not improved the outcomes of autoimmune cardiotoxicities. For stable patients in whom symptoms often appear while exercising or under stress but disappear after medication, diuretics and HF drugs should be provided until symptoms resolve. However, close monitoring needs to be done for arrhythmic patients with a low-threshold pacemaker to prevent complete heart blockage [26]. For unstable and highly symptomatic patients, immunotherapy should be stopped immediately and indefinitely until resolving IRAEs and associated cardiotoxicity [30]. Conventional cardiac failure therapies, such as β-blockers, calcium channel blockers, and renin-angiotensin system inhibitors, can be applied for severe and unstable patients who will show symptoms at any time, even at rest [6].

## 4. Preclinical Evaluation of mAbs in the Promotion of Cardiotoxicity

The manifestation of adverse cardiac effects in immunotherapy, both long- and short-term, is poorly understood from a mechanistic perspective. The use of whole-animal models in vivo and in vitro studies is insufficient and does not accurately reflect early and late cardiotoxic responses of the human myocardium. Most pharmaceutical drugs targeting cancer cells are currently being withdrawn based on adverse cardiotoxicity, even after extensive testing in animal models. This calls for developing reliable, sensitive, and clinically relevant models for efficacy and safety assessment of immune drugs for cancer. Human embryonic stem cells and hiPSC-CMs have recently been applied as an in vitro research tool for modeling diseases to elucidate drug-induced pathological cardiotoxicity mechanisms at the cellular level [51]. Such models can also be predictive of adverse effects of different immunotargets at the genome and epigenome levels. Today, hiPSC-CMs can be produced in less than 2 weeks, using chemically improved cardiac differentiation methods, as well as genetically modified, using modern genome editing technologies/tools [52]. Moreover, monoclonal hiPSC-CMs can be produced on a large scale for a rigorous and reproducible model of cardiotoxicity testing in vitro and on a smaller scale for patient-specific testing. The improved protocol to generate hiPSC-CMs without ethical challenges has given hope for developing targeted therapies for cancer patients in preclinical testing and may replace, or reduce, cardiac safety assessment assays in the future [53]. Human iPSC-CMs are authentically similar to cardiomyocytes based on the expression of ion-channels and sarcomere proteins, which helped in their initial identification and characterization. Moreover, they have similar electrophysiological properties, such as action potentials and calcium transients, linked to contractions, and, in particular, regular spontaneous beating. In addition, hiPSC-CMs at the cellular levels can mimic other cardiovascular disease phenotypes, such as long-QT syndrome, cardiomyopathies, and congenital heart diseases. These hiPSC-CMs are also a suitable model for studying the response of inotropic drugs, such as norepinephrine and beating rates upon electrical stimulation. Furthermore, hiPSC-CMs can be cultured as simple 2D or 3D organoids or developed into various complex genetic compositions that resemble adult cardiac tissues [52,53,54]. Cancer-associated cardiotoxicity has been characterized as acute, sub-acute, and chronic. Cardiac dysfunctions, such as pericarditis or myocarditis, along with abnormal electrical conduction and cardiomyopathies, are typically manifested as sub-acute cardiotoxicity (within 1 year of therapy). However, chronic toxicities have been reported in the range of 1.6% to 23% with late-onset occurring 10–20 years after therapy, owing to some new stress or previously injured cardiac substrate. Therefore, hiPSC-CMs are an excellent and attractive model for studying time-dependent effects of cancer immunotherapies since early cardiac markers could help detect and predict long-term cardiotoxicity [55]. However, further studies are needed to (1) determine the role of other cardiac cell types such as fibroblasts, endothelial cells, and atrial cells and (2) determine the requirement for additional systemic stimuli (hormonal, cardiotoxic metabolites) for cardiotoxicity not present in vitro. Also, to avoid false positives in new cancer immune-targeting drugs, traditional toxicity assays, or slow and deliberate dose-escalating clinical studies need to be performed along with hiPSC-CMs assays. Recently, Trastuzumab’s toxic effects have been confirmed using the hiPSC-CMs platform, showing that it blocked expressed human epidermal growth factor receptor and interfered with NRG1 signaling, leading to cardiotoxicity [56,57]. Furthermore, Trastuzumab decreased oxidative phosphorylation and glucose use, along with mitochondrial dysfunction in hiPSC-CMs, leading to cardiac toxicity that exhibited significant cardiac impairment but no cell death [58]. 

Although the use of hiPSC-CMs for high-throughput drug screening is becoming commonplace, it is more challenging to use hiPSC-CMs for hazard identification and risk assessment during drug development. Moreover, the need has arisen for robust reproducibility of fit-to-purpose preparation of hiPSC-CMs for clinically recognized gold standards [16,52,53,54,57]. These complex human-relevant preparations need to pass rigorous testing for reproducibility and justification for the higher costs. Currently, hiPSC-CMs are promising based on their supportive role in drug-induced cardiotoxicity safety testing during clinical trials.

## 5. Future Directions

**Figure 3:**
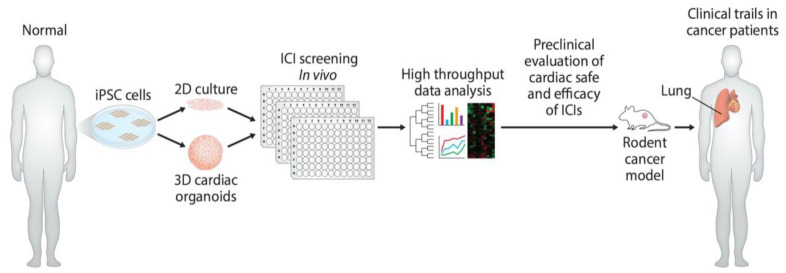
Development of preclinical models and determination of cardiac-safe ICIs. Diagram illustrates the use of hiPSC to develop in vitro human cardiomyocytes and cardiac organoids. These in vitro models can be used to screen monoclonal antibody-based ICIs for their cardiac toxicity. Further in vitro cardiac-safe ICIs can now be tested for toxicity in mouse tumor models and in cancer patients.

Even though the monitoring of cardiac output can now be achieved through noninvasive and invasive methodologies, such strategies are neither sufficient nor sensitive enough to discover early and late biomarkers responsible for ICI-induced cardiac toxicity. At the most basic level, iPSC-derived models are warranted to monitor cardiac function in response to monoclonal antibody-based immunotherapies (Figure 3). However, the ideal solution is to employ the H9C2 cell line, iPSC-derived organoid culture, and mouse models to validate data reproducibility. Coupling of high-volume omics data and molecular assessment of cardiac function will enable the identification of early and late biomarkers pursuant to the development of personalized therapies for ICI-induced cardiac toxicity, as well as advance the emerging cardio-oncology field.

## 6. Conclusions

The manifestation of adverse cardiac effects in monoclonal antibodies immunotherapies, both long- and short-term, is poorly understood from a mechanistic perspective. Using the new platforms and strategies for investigating cardiovascular toxicity by employing human-induced pluripotent stem cell-derived cardiomyocytes (hiPSC-CMs) and cardiac organoids, along with traditional biomarker detection and cardiovascular imaging would improve the safety and efficacy of cancer immunotherapies.

## Figures and Tables

**Table 1:** Different ICI-induced Cardiac Toxicities in Different Cancers.

Cancer Type	mAb (ICI)	Cardiac Toxicity	Reference
Melanoma	Ipilimumab and Nivolumab	Myositis with rhabdomyolysis, early progressive cardiac electrical instability and myocarditis	[37]
Non-small cell lung cancer	Nivolumab	Massive pericardial effusion	[38]
Hodgkin’s lymphoma	Pembrolizumab (KEYTRUDA)	Myocardial infarction, pericardial effusion, pericarditis, arrhythmia and cardiac tamponade	[39]
Kidney cancer	Nivolumab	Myocarditis	[40]
Advanced urothelial carcinoma	Pembrolizumab and Atezolizumab	Immune myocarditis	[41]
Merkel cell carcinoma	Nivolumab	Myocarditis	[42]
Squamous cell neck cancer	Nivolumab	Myocarditis and ventricular arrhythmia	[42]
Squamous cell neck cancer	Pembrolizumab	Cardiac failure	[42]

## Data Availability

All the data presented in this study are included in this article.

## Abbreviations

ACT	Adoptive cell therapy
TIL	Tumor-Infiltrating Lymphocyte
TCR	T-Cell Receptor
CAR	Chimeric Antigen Receptor
hiPSC-CMs	human-induced pluripotent stem cell-derived cardiomyocytes
CTLA-4	Cytotoxic T-lymphocyte-associated antigen 4
ASE	American Society of Echocardiography
EACVI	European Association of Cardiovascular Imaging
IRAEs	Immune-related adverse events
IMARs	Immune-related adverse reactions
ICI	Immune checkpoint inhibitors
LVD	Left ventricular dysfunction
HFpEF	Heart failure with preserved ejection fraction
PD-1	Programmed cell death protein-1
PD-L1	Programmed death ligand-1
